# How does the pelvic floor respond to modulations in trunk pressure induced by a variety of voicing tasks? A cross‐sectional, observational study

**DOI:** 10.14814/phy2.70090

**Published:** 2024-10-18

**Authors:** Aliza Rudavsky, Linda McLean

**Affiliations:** ^1^ Department of Kinesiology Pennsylvania State University University Park Pennsylvania USA; ^2^ School of Rehabilitation Sciences University of Ottawa Ottawa Ontario Canada

**Keywords:** pelvic floor, stress urinary incontinence, transperineal ultrasound, trunk pressure, voicing

## Abstract

The pelvic floor responds to changes in trunk pressure, elevating during low‐pressure exhale and descending during high‐pressure exhale. Voicing occurs during exhalation, spanning low‐to‐high trunk‐pressure, yet it is unknown how voicing affects the pelvic floor. The aim of this study was to quantify pelvic floor response to voicing and identify if there are differences for women with stress urinary incontinence. We hypothesized that shouting would cause pelvic floor descent, with greater magnitude for incontinent women. Sixty women (38 incontinent, 22 continent) performed four voicing tasks (counting to “4” in speaking/shouting/low‐pitch/high‐pitch voice) while transperineal ultrasound measured changes in pelvic floor morphology. ANOVA compared variance of responses to voicing and *t*‐tests compared groups. Bladder neck height shortened, levator plate length increased and levator plate angle decreased more during shouting compared to speaking; consistent with pelvic floor straining. There were no differences for high versus low pitch‐voicing and small group differences based on continence status. Voicing causes pelvic floor muscles to strain, with greater strain during shouting. Changing vocal pitch does not affect pelvic floor morphology and incontinent women had slight differences from continent women. Voicing may be a safe way to lengthen the pelvic floor without provoking incontinence.

## 1 INTRODUCTION

Stress urinary incontinence (SUI) affects 34% of women between the ages of 18–60 (Nygaard et al., 2005), One in three women over 40 report leaking associated with quick bursts of laryngeal pressure—activities such as laughing, sneezing or coughing (Coyne et al., 2009). Other voicing activities such as speaking, singing, shouting, and grunting also require changes in laryngeal pressure. These activities are not typically considered in SUI research but may be provocative for women with SUI as it is unknown what motion these variable laryngeal pressures cause in the pelvic floor. Preliminary research has shown that healthy people strain their pelvic floor during most voicing tasks (Rudavsky & Turner, 2020). As such, voicing activities may impact pelvic organ support and urinary continence control.

The trunk can be modeled as a pressure cannister, with a cylinder formed by the rib cage, spine and abdominal muscles. The cannister is bound by the larynx at the top, the pelvic floor at the bottom, and the respiratory diaphragm creating a regulator in the middle. During activities associated with low thoracic‐abdominal pressure, such as natural breathing, all three structures move in a phase‐locked coordination to descend on inhalation and ascend on exhalation (Harper et al., 2013; Talasz, Kremser, et al., 2011). During tasks that induce very high‐pressures, such as forced exhalation and coughing, the pelvic floor reverses its motion, descending on exhalation while the diaphragm elevates (Rudavsky & Turner, 2020; Talasz et al., 2010, 2021). Thus, somewhere along the pressure continuum between low‐pressure and high‐pressure tasks, the pelvic floor coordination switches from ascending on exhalation to descending on exhalation. This transition has not been studied but is important to understand in order to identify daily activities that generate laryngeal pressures that may either provoke pelvic floor symptoms or could potentially be employed to improve pelvic floor function.

Voicing occurs when the vocal folds at the top of the trachea are adducted and air pressure from the lungs builds up below the folds causing vibration at the vocal folds or sound waves. Modulating the air pressure from the lungs can influence the frequency of the vocal fold vibrations (the pitch of the sound) or the amplitude of the sound waves (the loudness of the sound) (Titze, 1989). Voicing is an activity that is done only on exhalation and under pressures ranging from low to high, depending on the specific task demands. Because of the variability of air flow and thus abdominal‐thoracic pressure demand during voicing, it is unknown if the pelvic floor responds more similarly to natural exhale (elevate) or forced exhale (descend), and the response may depend on vocal pitch and/or loudness as they are also influenced by modulations in trunk pressure. The aim of this study was to measure transient changes in pelvic morphology, specifically pelvic floor length, levator plate angle (LPA)/anorectal junction motion, and bladder neck (BN) descent, induced during voicing tasks performed at different pitches (deep, high) and volumes (quiet, loud), and to determine whether changes differ between continent women and those with SUI.

We hypothesized that there would be evidence of pelvic floor strain (BN descent, levator plate elongation, LPA reduction) during voicing, which would be greater during a shouting compared to a speaking volume because louder voicing requires more pressure in the trunk, and that differences would be observed between voicing at high compared to deep vocal pitch because of differences in vibration frequency. Further, we hypothesized that women with SUI would demonstrate greater pelvic floor strain in response to voicing compared to continent women.

## 2 METHODS

### 2.1 Study design

This was an observational, case–control study. The protocol was approved by the Institutional Review Board at the Pennsylvania (Penn) State University as being compliant with national standards for the ethical conduct of research.

Women over the age of 18 years were recruited from the local Penn State University and State College, PA communities. Exclusion criteria included: women who were pregnant or less than one‐year post‐partum, chronic respiratory conditions (including asthma, Chronic Obstructive Pulmonary Disease), neurologic disorders (such as Parkinson's Disease or Multiple Sclerosis), history of major gynecologic surgery (i.e., hysterectomy), persistent cough, vocal or respiratory dysfunction, or moderate to severe back pain in the previous 12 weeks. Participants were cohorted into the symptomatic group based on their self‐reported symptoms of SUI in response to the following question: “Do you leak urine with coughing, sneezing, laughing, exercising?” Answer options include: 0 = Never, 1 = Occasionally (<1×/week), 2 = Frequently (≥1×/week), and 3 = Daily (Baessler et al., 2010). The groups were then dichotomized into continent controls who reported never leaking, and the SUI group who reported occasional, frequent or daily leakage. Any other pelvic floor symptoms were recorded by their responses to the Australian Pelvic Floor Questionnaire (APFQ) which asks about bowel, bladder, prolapse and sexual symptoms (Baessler et al., 2010). Background information on parity history was also collected. All participants provided written, informed consent prior to undergoing any aspect of the study protocol.

### 2.2 Clinical tests

Respiratory diaphragm muscle function is important for both voice production and pelvic floor function (Bordoni & Zanier, 2013). Because of the anatomy of the diaphragm, contraction (which is related to inhalation function) causes expansion of the rib cage (Bordoni et al., 2016; Bordoni & Zanier, 2013; Troyer & Wilson, 2016). To assess diaphragm function, participants had lower ribcage excursion measured during a full breath‐cycle (Troyer & Wilson, 2016). Excursion was measured using a cloth tape measure around the lower ribcage, placed two finger‐widths below the xyphoid process (Bockenhauer et al., 2007).

Because of COVID‐19 related laboratory and research restrictions at the time of data collection, the study group was limited in the amount of time spent in a private room with the participants and therefore there was no physical examination of pelvic floor muscle function. The self‐administered APFO was used to evaluate global pelvic floor symptoms. This questionnaire evaluates the domains of bowel, bladder, prolapse and sexual function and has been validated for routine clinical and research assessment (Baessler et al., 2010).

### 2.3 Trans‐perineal ultrasound imaging

A 5‐MHz curved array ultrasound transducer (Phillips iU22 MATRIX, Phillips Healthcare, Andover, MA, USA), was used to image pelvic morphology. Due to complications of the COVID‐19 pandemic, image collection was done by two different expert sonographers, as the initial sonographer had to leave the study team before its completion. Each sonographer had over 10 years of experience in gynecologic ultrasound and received 10 h of hands‐on training using the specific techniques described below.

Participants emptied their bladder prior to beginning data collection. Participants donned a gown and removed undergarments. Ultrasound imaging was performed in standing because it is a more functional position than supine, and integrates the effect gravity on tissue morphology and muscle function (Dietz & Clarke, 2001; Frawley et al., 2006). During all ultrasound scans, participants stood with their back and pelvis leaning against a wall to minimize postural sway, and with their lumbar spine/pelvis in a neutral position, their knees bent comfortably, and their feet shoulder width apart.

Ultrasound gel was applied to the transducer, which was then covered with a sterile probe cover and more ultrasound gel. The sonographer held the transducer on the perineum, oriented in the mid‐sagittal plane to enable concurrent visualization of the public symphysis (PS), the BN and the anorectal angle (ARA) within the imaging frame. For each task, a cine loop was recorded from the rest position to the end of the task.

### 2.4 Tasks

Six tasks were performed, with three trials of each task performed in a semi‐randomized order to control for bladder filling or other systematic effects. The first two tasks were specifically performed with instruction and cuing to generate either a maximum pelvic floor maximal voluntary contraction (MVC) or a maximal effort straining maneuver, and the participants were encouraged to use the ultrasound monitor for feedback on their performance during the task (Dietz et al., 2001). Using the screen, pelvic floor elevation or descent was observed and confirmed visually prior to commencing data collection. If participants had difficulty correctly performing the tasks, different verbal cues were used on an individual basis. For the MVC, the initial cue was to “cut off the flow of urine mid‐stream and try to suck it back in” or “suck a tampon or menstrual cup in.” For the strain task, the cue was to “bear down” or “try to push a tampon or menstrual cup out.” Participants did not have to hold the tasks and were encouraged to lift or lower their pelvic floor as much as possible then release.

The other four tasks were voicing tasks which involved counting from one to four on one continuous exhalation. Because higher pitches and louder volumes require more laryngeal and thoracic pressure (Sundberg et al., 1993; Titze, 1989), the voicing tasks included (1) a deep pitch at a speaking volume, (2) a deep pitch at a shouting volume, (3) a high pitch at a speaking volume, and (4) a high pitch at a shouting volume. Three trials of each task were performed in a randomized order. Participants were cued only on the voice task; no instruction was given regarding pelvic floor muscle contraction/relaxation during the task, and participants were not permitted to look at the ultrasound monitor during the voicing tasks.

### 2.5 Data processing

**FIGURE 1:**
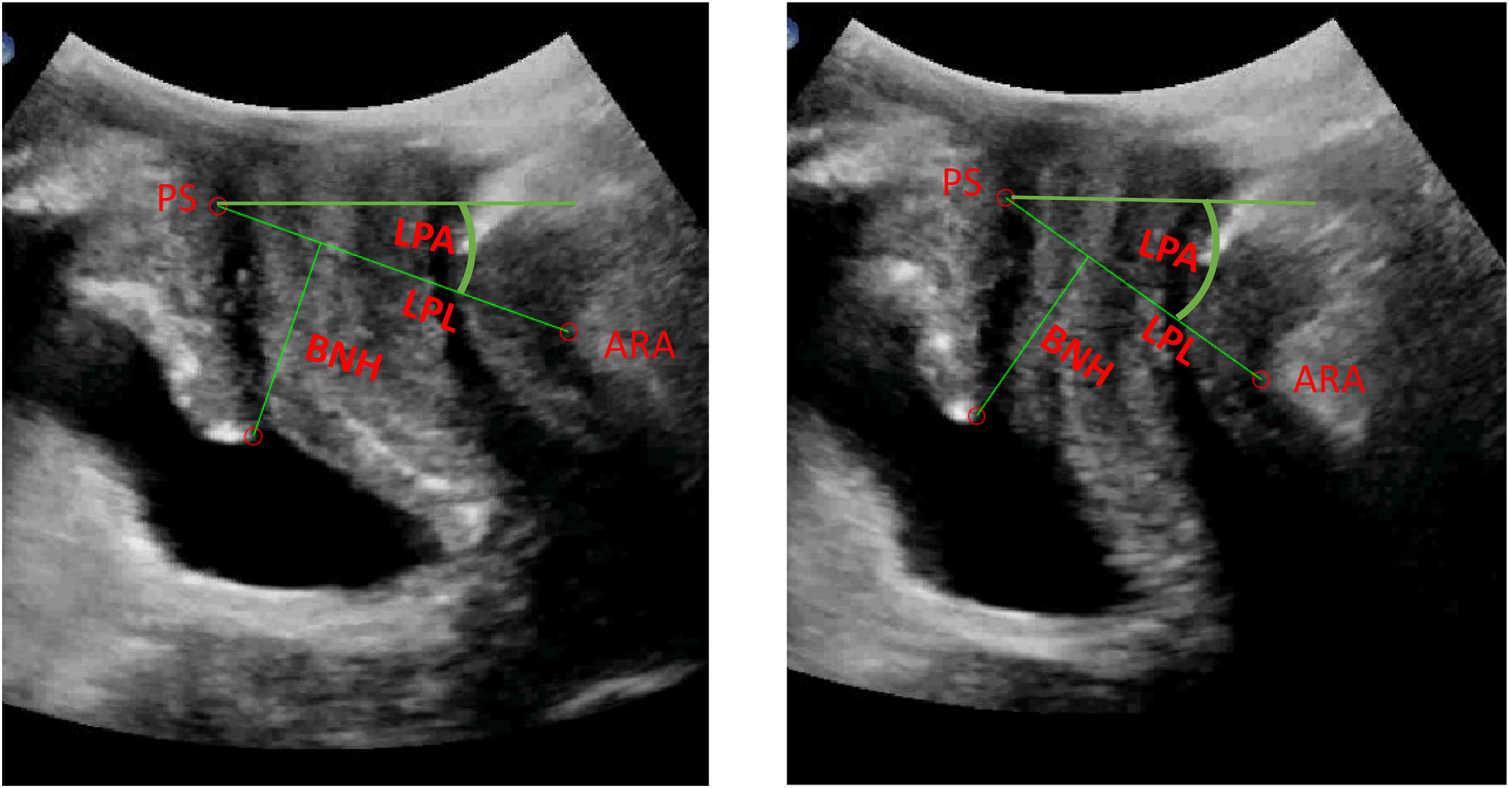
Transperineal ultrasound measure of pelvic muscle morphometry during rest (left) and max voluntary contraction (right): Bladder neck height (BNH), Levator plate length (LPL), and Levator plate angle (LPA), Pubic Symphysis (PS) and Anorectal Angle (ARA) are labeled on the images.

All measurements were completed by a separate rater who was blinded to whether or not participants experienced urinary incontinence. From each ultrasound cine loop recorded during each task, the rater took measures on a frame at the beginning of the video that best represented the rest position, and on a frame that best represented the maximal displacement (Figure 1). The UROKIN method Czyrnyj et al. (2018) using MATLab (Mathworks, Natick, MA, USA) was used to process images, which involved identifying the most posterior/inferior point on the pubic symphysis (PS) and the apex of the ARA on each frame. The program connected these two points with a line and measured the levator plate length (LPL). A change in the LPL reflects morphological changes of the anterior sling of the levator ani muscle (pubovisceral), with anterior motion of the ARA relative to the PS reflecting levator ani contraction (reduction in the LPL) and posterior motion of the ARA relative to the PS reflecting strain (lengthening of the LPL) (Muro & Akita, 2023; Muro et al., 2014). Next, the BN was identified and the program calculated the perpendicular distance from the BN to the line connecting the ARA to the PS, which is the LPL (Figure 1). The length of the second line was used to measure BN height with respect to the levator plate. Last, the LPA was calculated as the angle between the levator plate and the horizontal, which is considered to be a measure of cranial and caudal displacement of the posterior sling of the levator ani (iliococcygeus) which moves the anorectal junction caudally or cranially (Muro & Akita, 2023; Muro et al., 2014). Pelvic floor landmark displacement was calculated through using the UROKIN method by taking the difference between the rest position and at maximal excursion for each measure during each task. For each measure and task, the average value over the three trials was retained for analysis. For the voicing tasks, morphological change was normalized to the morphologic change observed during the maximum strain task, and reported as percent of maximal strain (%strain).

### 2.6 Statistical analysis

Statistical analyses were performed using SPSS Statistics for Windows, Version 28.0.1.0 (IBM Corp., Armonk, NY, USA). Parametric descriptive statistics (i.e., total displacement and %strain) were presented for each voicing task. For the voicing tasks, separate analysis of variance (ANOVA) models were used to compare the effect of vocal pitch (deep, high) and volume (quiet, loud) on each outcome (change in LPL, change in BN height, change in LPA) using both normalized (%strain) and non‐normalized values across the entire sample. To compare symptom effects on BNH, LPL and LPA morphology changes during voicing, 3‐way ANOVA was used with fixed factors of symptoms, vocal pitch and vocal volume, as well as all two‐ and three‐way interaction effects. One‐sided, Welch's *t*‐test was used to compare pelvic floor morphology changes in response to contraction, strain and voicing between continent women and those with SUI.; *α* was set at *α* = 0.05.

### 2.7 Sample size

As no previous study has measured changes in pelvic floor morphometry during voicing, we assumed a normal distribution would occur across a random sample of women. Using G*power (3.1.9.7) and based on a moderate effect with alpha = 0.05 and power = 0.8, we estimated that 59 participants would provide adequate power.

## 3 RESULTS

Sixty women between the ages of 20 and 68 (mean age 40) years participated. Table 1 includes participant demographic characteristics and outcomes from the clinical tests. Participants had a wide range of pelvic floor symptoms, and those with SUI had a wide range of symptom severity. Total scores on the APFQ, and Bladder construct of the APFQ were higher among those with SUI than those without and more participants in the SUI group were parous compared to controls.

**TABLE 1:** Participant demographic information and functional test outcomes.

Category	Grouping	Overall sample (*n* = 60)	SUI (*n* = 38)	Control (*n* = 22)
Parity status	Nulliparous	15 (25.0%)	5 (13%)*	10 (45%)*
	Parous	45 (75.0%)	33 (87%)	12 (55%)
Number of vaginal deliveries	0	21 (35.0%)	9 (24%)	12 (55%)
	1	15 (25.0%)	12 (32%)	3 (14%)
	2	18 (30.0%)	14 (37%)	4 (18%)
	3	2 (3.3%)	1 (3%)	1 (5%)
	4	4 (6.7%)	2 (5%)	2 (9%)
Number of cesarian deliveries	0	49 (81.7%)	29 (76%)	20 (91%)
	1	7 (11.7%)	7 (18%)	0 (0%)
	2	2 (3.3%)	1 (3%)	1 (5%)
	3	2 (3.3%)	1 (3%)	1 (5%)
Ribcage excursion (cm)	Mean (SD)	5.8 (2.2)	5.55 (2.33)	6.27 (1.96)
APFQ total (0–40)	Mean (SD)	4.3 (3.1)	4.95 (3.55)*	3.2 (1.88)*
APFQ bladder (0–10)	Mean (SD)	1.3 (1.1)	1.65 (1.07)*	0.7 (0.8)*
APFQ bowel (0–10)	Mean (SD)	1.6 (1.0)	1.6 (1.0)	1.6 (0.98)
APFQ prolapse (0–10)	Mean (SD)	0.4 (1.1)	0.6 (1.4)	0.2 (0.4)
APFQ sexual (0–10)	Mean (SD)	1.0 (1.3)	1.1 (1.4)	0.7 (0.9)
SUI severity	None	22 (36.7%)	0 (0%)	22 (100%)
	Occasional	29 (48.3%)	29 (76%)	0 (0%)
	Frequent	6 (10.0%)	6 (16%)	0 (0%)
	Daily	3 (5.0%)	3 (8%)	0 (0%)

Abbreviations: APFQ, Australian Pelvic Floor Questionnaire; SUI, Stress Urinary Incontinence.

* Indicates statistically significant difference between SUI and control group (*p* < 0.05).

### 3.1 Response to voicing parameters

**FIGURE 2:**
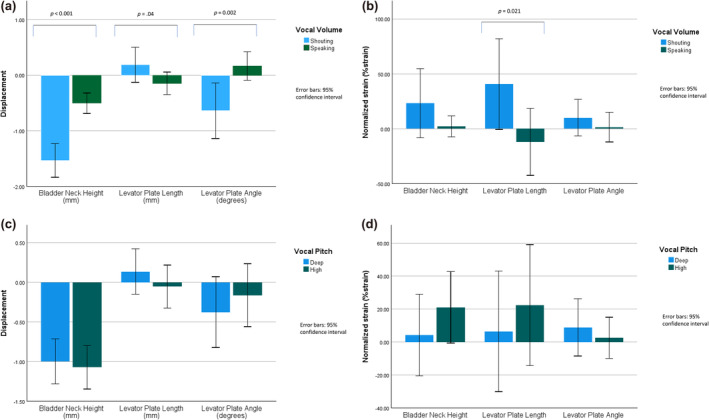
Pelvic Floor landmark displacement in response to vocal‐volume changes (a). Normalized displacement (%strain) of pelvic floor landmarks in response to vocal‐volume changes (b). Pelvic Floor landmark displacement in response to vocal‐pitch changes (c). Normalized displacement (%strain) of pelvic floor landmarks in response to vocal‐pitch changes (d).

Pelvic floor landmark displacement observed across all tasks is presented in Figure 2a–d. To interpret the figures, a negative BN height displacement is consistent with the BN lowering during the voicing task (as seen during a strain), a positive displacement of LPL is consistent with levator plate elongation during the voicing task (as seen during a strain), and negative displacement of LPA is consistent with the ARA lowering during the voicing task (as seen during a strain). Considering absolute landmark displacement, there was greater pelvic floor strain during shouting compared to speaking. Specifically the BN descended [shouting: −1.53 ± 1.7 mm; speaking: −0.50 ± 1.0 mm, *t*(238) = 5.75, *p* < 0.001, SE = 0.18], the LPL lengthened [shouting: 0.19 ± 1.7 mm; speaking, −0.14 ± 1.1 mm, *t*(238) = −1.76, *p* = 0.04, SE = 0.19], and the LPA became larger [shouting: −0.64 ± 2.76 degrees; speaking: 0.16 ± 1.4 degrees, *t*(238) = 2.83, *p* = 0.002, SE = 0.28]. When normalized to maximum strain, the relative displacement of the LPL also suggested greater strain during shouting [shouting: 39.3 ± 217.3%; speaking: −11.3 ± 160.7%, *t*(238) = −2.05, *p* = 0.021, SE = 24.7], but there were not differences between shouting and speaking observed in the relative displacement of the BN or LPA. There were no differences observed in absolute or relative pelvic floor landmark motion observed between deep versus high vocal pitch.

### 3.2 The effect of continence status on pelvic floor morphology during contraction, strain and vocal tasks

**FIGURE 3:**
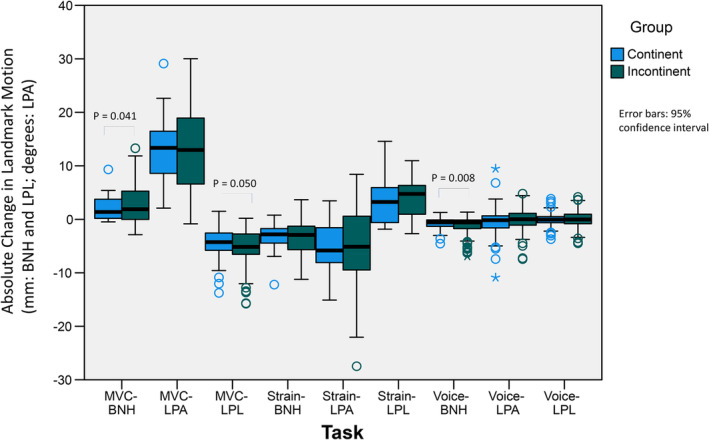
Mean response to tasks by continence status. BNH, Bladder Neck Height; LPA, Levator Plate Angle; LPL, Levator Plate Length; MVC, Maximal Voluntary Contraction.

During the MVC task, LPL shortened more in the group of women with SUI (−5.8 ± 4.3 mm) compared to those who were continent (mean difference = −4.9 ± 3.7 mm), *t*(204) = 1.65, *p* = 0.05, *d* = 0.22, approaching statistical significance, and the BN elevated more in the women with SUI (2.9 ± 4.0 mm) compared to those who were continent (2.1 ± 2.4 mm), *t*(238) = −1.751, *p* = 0.041; *d* = −0.22. No differences were observed in the LPA between women with SUI and controls during MVC (continent women mean change = 13.1 ± 6.5 degrees, SUI mean change = 13.6 ± 8.3 degrees, *t*(217) = −0.549, *p* = 0.71). During maximal straining efforts, there were no differences in the extent of changes in pelvic morphology observed between continent women and those with SUI. [BNH: continent (−3.3 ± 2.8 mm) versus SUI (−3.4 ± 3.3 mm), *t*(205) = 0.465, *p* = 0.32; LPL: continent (4.0 ± 4.7 mm) versus SUI (3.8 ± 3.6 mm) *t*(145) = 0.358, *p* = 0.361; LPA: continent women (−5.5 ± 5.34 degrees) versus SUI (−5.3 ± 7.7 degrees), *t*(230) = −0.21, *p* = 0.581] (Figure 3).

During voicing tasks, there were no significant two‐ or three‐way interactions among SUI status, pitch and volume, yet BNH was lower during voicing among those with compared to those without SUI (*p* = 0.024). there were no significant interactions nor main effects in the 3‐way ANOVA (continence status, pitch, volume) for LPA or BNH.

## 4 DISCUSSION

To our knowledge, this is the first study to investigate transient changes in pelvic floor morphology induced by voicing tasks. Among females, voicing tasks induce BN descent, levator plate strain and enlargement of the ARA as described by reduction in the LPA, modulated by vocal *loudness* but not by vocal *pitch*. Further, we found that women *with* SUI elevated their BN and shortened their levator plate more than continent women when performing a maximal‐effort voluntary contraction, but they did not demonstrate greater BN descent, levator plate lengthening or LPA increase during the maximum effort straining task. This could be due to women with SUI employing a regular pelvic floor lift or concentric contraction to reduce episodes of leakage. Greater BN descent during voicing was observed among those with SUI compared to continent women, however the extent of this descent was very small, in the order of 1 mm, and thus may have limited clinical relevance.

While there is limited prior literature on the impact of voicing tasks on transient changes in pelvic floor morphology, our findings are consistent with a previous pilot study that showed pelvic floor strain during voicing (Rudavsky & Turner, 2020). During shouting, BN descent reached 34.4% of the peak displacement observed during maximal effort straining, yet this amounted to only a small absolute displacement in the order of 1 mm. Similarly, during shouting, the levator plate lengthened significantly compared to speaking, but again the length change was very small (<0.5 mm). The LPA, which reflects the vertical position of the ARA relative to horizontal, changed by less than 1 degree. While these differences in pelvic floor morphology during voicing are very small, they all point to strain, with greater strain induced by shouting compared to speaking. Yet the clinical relevance of these small changes must be considered, especially in light of the lack of difference observed between those with SUI and continent women. Perhaps these tasks which elicit bursts of laryngeal pressure are not strong enough to be problematic in women with SUI and could actually be used to build tolerance for higher, more provocative pressure‐generating tasks (i.e., coughing, sneezing, laughing).

It is not surprising that changes in vocal pitch had no effect on the extent of pelvic floor strain during voicing. Increasing vocal loudness requires an elevation in thoracic pressure, which can lead to descent of the pelvic organs, whereas increasing vocal pitch requires an increase in the frequency of vocal fold vibration, which predominantly happens through lengthening the vocal folds (Holmberg et al., 1989; Rudavsky & Turner, 2020; Zhang, 2016). The pelvic floor response observed during deep and high pitch voicing had high variance, which may have been due to the sample not being trained vocalists, which also may have impacted our capacity to observe differences in pelvic floor strain induced by different vocal pitches.

It is not possible to know if the pelvic floor muscles were active during the voicing tasks as we did not, record electromyographic activity during the protocol. As such, it is not clear whether the pelvic floor muscles contracted isometrically or eccentrically to limit strain during voicing, or whether the pelvic floor muscles were lengthened passively. While the changes in morphology are small, the findings of this study identify the transition from speaking to shouting as the reversal of the pelvic floor motion between quiet breathing and forceful exhalation.

No direct comparisons that can be made between findings on the voicing tasks studied here and the literature. A small MRI study on 8 healthy individuals (Talasz, Kremser, et al., 2011) showed that, in the supine position, there is a phase‐locked coordination between the pelvic floor (pubococcygeal line and puborectalis muscle sling) and respiratory diaphragm such that on natural breathing both structures moved caudally on inhalation and cranially on exhalation. The authors reported that forced exhalation resulted in cranial motion of the pelvic floor, which is not what we found, however, the start position for the forced exhalation task was at maximal inhalation, thus in that study, the pelvic floor likely began in a position that was caudal relative to its resting position (Talasz, Kremser, et al., 2011). Further, findings may depend on glottis state (open, closed), specific pressure demand, or body position relative to gravity.

It has been suggested that a pelvic floor muscle contraction normally precedes or occurs co‐incident with tasks that cause a rise in intra‐abdominal pressure (Smith et al., 2007). Contrary to this expectation, among 149 healthy, nulliparous women, De Jong, et al. observed that the BN did not move cranially in 92% of women during coughing and in 70% or women during forced exhalation (de Jong et al., 2023). While the authors did not report on caudal landmark displacement, their findings are consistent with our findings that the BN descended, the LPL shortened and the LPA reduced during voicing, particularly with shouting. Similarly, through visual observation of the perinium, Talasz et al. reported that 24 of 40 (60%) asymptomatic women contracted their levator ani at the initiation of a forced exhalation task while the perineum descended during coughing in 36 (90%) of them (Talasz et al., 2010). Talasz et al. suggested that an externally observable caudal descent of the perineum (bulge) during forced exhalation and coughing reflects incorrect co‐ordination. Yet the findings of the current study suggest that a caudal descent of the pelvic organs may be a normal response to increased laryngeal‐induced intra‐abdominal pressure, likely to accommodate quick and high bursts of thoracic‐abdominal pressure. That is, women may rely more on passive support from connective tissues than on active support of the pelvic organs through PFM contraction.

The finding that women with SUI lift the BN and shorten levator plate more than continent women is in contrast to what was reported in a 2015 systematic review, which suggested that continent women lift their pelvic floor more cranially during MVC and incontinent women lower it more caudally during Valsalva (Leitner et al., 2015). However, the review also reported that there were inconsistent findings among studies. Some have found no differences in pelvic floor displacement between women with and without SUI during contraction (Thompson et al., 2007), while others have found larger ARA and BN lift during contraction among those with SUI compared to those who are continent (Czyrnyj et al., 2018).

One major difference between the previously described studies reporting on forced exhalation and the current study reporting on voicing is the function of the glottis and vocal folds. During inhalation and exhalation, the vocal folds are abducted and the glottis is open for air to pass through. During a strain task, the glottis is closed and the vocal folds are fully adducted, preventing air from passing (Talasz, Kofler, & Lechleitner, 2011). For voicing, however, the vocal folds are adducted but not completely closed as the air from the lungs is forced cranially. When the subglottal pressure from under the vocal folds overcomes the resistance from the adducted folds, it causes a vibration in the folds which results in phonation or sound production (Esling, 2006). This may be important for pelvic floor strain because voicing is a way of modulating the release of high trunk pressure (i.e., shouting) and may facilitate controlled descent of the pelvic floor, whereas uncontrolled rises in pressure (coughing, sneezing, vomiting) may provoke uncontrolled pelvic floor strain which may lead to leakage. There may be value in using progressively louder voicing as a means of building pelvic floor muscle control such that these muscles learn to respond to controlled, forceful exhalations, with eventual transfer to uncontrolled pressures.

### 4.1 Strengths and limitations

There are several notable limitations to this study, one key issue was that, due to Covid‐19 restrictions in place at the time of data collection, we were not permitted to perform a physical pelvic floor muscle evaluation. While we could not confirm through palpation that all participants were able to contract their pelvic floor voluntarily, we did observe landmark motion on ultrasound imaging during MVC that suggests that all participants were able to contract their pelvic floor muscles, at least to some extent.

We used the APFO to classify participants as having SUI or not. However, this approach is valid as SUI is defined as a subjective experience, and self‐report is clinically relevant. That said, we had no objective measure of symptom severity. Additionally, as other pelvic floor symptoms were not considered exclusion criteria and we relied on subjective reporting of symptoms versus pelvic exam, participants may have had additional pelvic disorders that were not recorded.

All participants were required to wear masks throughout the data collection session, which meant that we could not measure vocal pitch or loudness objectively. Masking may also have provided additional vocal pressure resistance that was not uniform for each participant, and may have depended on the type of mask they wore.

While all image processing of ultrasound videos was done by the same rater, the scans themselves were performed by two different raters who used the same machine. While unlikely, it is possible that this affected the ultrasound measurements.

## 5 CONCLUSION

This is the first study to measure pelvic floor strain during voicing tasks in asymptomatic women and those with SUI. While the observed changes were very small, we found that pelvic floor landmarks change in the same pattern as straining: the BN descends, the LPA reduces, and the levator plate lengthens during voicing, and that vocal loudness increases the magnitude of these changes. Vocal pitch does not affect pelvic floor motion. Women with SUI showed greater BN descent during voicing, and greater BN elevation and levator plate shortening during contraction than continent women. In terms of clinical implications, it appears that transitioning from a speaking to a shouting voice can facilitate a change in pelvic floor length in both symptomatic and asymptomatic women, with greater BN descent observed among symptomatic women. These tasks appear to be small enough that they are unlike to be symptom provoking but have the potential to be used clinically to gradually build tolerance to higher, more provocative loads.

## AUTHOR CONTRIBUTIONS

**Aliza Rudavsky:** Project development, data collection and analysis, manuscript preparation. **Linda McLean:** Project development, data analysis, manuscript preparation.

## FUNDING INFORMATION

This research was funded by the Foundation for Physical Therapy Research‐ Pelvic Health Grant, 2019.

## ETHICS STATEMENT

This study received ethical approval from the Institutional Review Board at the Pennsylvania State University.
